# Clinical impact of bladder biopsies with TUR-BT according to cytology results in patients with bladder cancer: a case control study

**DOI:** 10.1186/1471-2490-10-12

**Published:** 2010-06-30

**Authors:** Masashi Matsushima, Eiji Kikuchi, Masanori Hasegawa, Kazuhiro Matsumoto, Akira Miyajima, Mototsugu Oya

**Affiliations:** 1 Department of Urology, Keio University School of Medicine, Tokyo, Japan

## Abstract

**Background:**

There seems to be no consensus concerning taking bladder biopsies during transurethral resection of bladder tumor (TUR-BT). We investigate the clinical significance of bladder biopsy with TUR-BT and the relationship between urinary cytology and the biopsy results.

**Methods:**

We reviewed a total of 424 patients with non-muscle invasive bladder cancer treated with TUR-BT between 1998 and 2005. Of the total, 293 patients also underwent a bladder biopsy. Biopsies from suspicious-appearing urothelium (N = 59) and those from normal-appearing urothelium (N = 234) were evaluated separately.

**Results:**

Bladder cancer was observed in 23 cases (39.0%) who underwent a biopsy of suspicious-appearing urothelium. Among these 23 cases, 9 cases with visible tumor resection had carcinoma in situ (CIS) only in the biopsies from suspicious-appearing urothelium. Urinary cytology was negative in 3 of the 9 cases. Bladder cancer was observed in 26 cases (11.1%) who underwent a biopsy of normal-appearing urothelium. Of them, 5 cases with visible tumors had CIS only in the multiple biopsies from normal-appearing urothelium. Urinary cytology was positive in all of the 5 cases. No upstaging or upgrading cases were found in these patients by the addition of these two types of biopsy. Furthermore, therapy was not altered in these patients. With or without bladder biopsy was not a significant factor for tumor recurrence in either the univariate or multivariate analysis.

**Conclusions:**

Based on the results, it is concluded the multiple biopsies from normal-appearing urothelium are not necessary in patients with negative cytology results because of the low detection rate and lack of influence on therapeutic decisions. Meanwhile, biopsy of suspicious-appearing urothelium is needed in patients with negative cytology results in order to detect CIS due to staging properties. This result supports a recent EAU guideline.

## Background

Bladder cancers are classified as being non-muscle-invasive or muscle invasive according to their histological appearance. Non-muscle-invasive bladder cancers account for about 70 to 80% of all bladder cancers[1-4]. Non-muscle-invasive tumors have a heterogeneous clinical course ranging from low-grade Ta tumors to highly malignant Tis and T1 lesions[5-7]. Low-grade carcinomas rarely exhibit progression. However, high-grade carcinomas do recur within a short period and sometimes invade the muscle layer, necessitating a change in treatment[7]. The primary approach for Ta and T1 tumors is transurethral resection of bladder tumor (TUR-BT). The main problem after the initial treatment of non-muscle-invasive bladder cancers is the high recurrence rate. Approximately 40 to 80% of Ta-T1 tumors recur after the initial treatment[2]. Many prognostic factors, such as tumor stage, tumor grade, multiplicity, concomitant carcinoma in situ (CIS), and tumor size have been proposed to affect tumor recurrence of non-muscle-invasive bladder carcinomas[6,8-11]. Some reports have suggested the importance of biopsy of bladder mucosa from normal appearing urothelium or from reddish areas at the time of initial treatment for bladder carcinoma as an additional biopsy might improve the diagnostic accuracy for bladder cancer staging and alter the therapeutic option for the tumor[7,8,10,12,13]. Meanwhile, an EAU guideline suggested that routine random biopsy is not advised if bladder mucosa has a normal aspect and urine cytology is negative. Furthermore, other investigators have suggested the possibility that bladder biopsies induce implantation of tumor cells at the biopsied mucosal site[6,7,10,14-17]. Still, there seems to be no consensus concerning taking bladder biopsies during TUR-BT[2,4,10].

In the present study, we evaluated 1) the clinical significance of bladder biopsies from suspicious-appearing urothelium and multiple biopsies from normal-appearing urothelium separately, 2) not only tumor up-staging but also up-grading of these biopsies, 3) the relationship between urinary cytology and biopsy results, 4) its impact on further treatment decision-making, and 5) whether or not additional bladder biopsies promote the intravesical recurrence of bladder carcinoma.

## Methods

Between 1998 and 2005, 424 patients with non-muscle invasive bladder cancer were treated with TUR-BT at Keio University Hospital in Tokyo. The mean age of the patients was 68.7 ± 0.57 (25-92) years old. The patients consisted of 349 men and 75 women. The median follow-up period was 3.6 ± 0.1 years. Bladder biopsies in 59 patients had been performed from reddish or mossy areas apart from the main tumor and edema of the bladder mucosa (biopsy from suspicious-appearing urothelium). Multiple biopsies of apparently normal mucosa were performed systemically in 234 patients (multiple biopsies from normal-appearing urothelium) during TUR-BT. Thirty-four patients underwent both a biopsy from suspicious-appearing urothelium and multiple biopsies from normal-appearing urothelium. Thus, a total of 293 patients underwent bladder biopsies. Basically, all patients with reddish or mossy areas apart from the main tumor and edema of the bladder mucosa underwent biopsy from suspicious-appearing urothelium. Meanwhile, multiple biopsies from normal-appearing urothelium were performed based upon the decision of the attending doctor. Multiple biopsies from normal-appearing urothelium were carried out to examine the following 6 cystoscopically normal-appearing bladder mucosal sites: (1) bladder floor, (2) right wall, (3) left wall, (4) dome, (5) posterior wall, and (6) prostatic urethra (in males) or bladder neck (in females).

The study was conducted subject to the guidelines of the Declaration of Helsinki and had no influence on the treatment of the patients. Keio university hospitals ethical committee granted ethical approval for the study. We obtained the patients' informed consent to carry out the surgery including their approval for potential use of their anonyms medical data from our data base for research and audit purposes.

Patient characteristics, including age, sex, tumor grade, initial number of tumors, pathological stage, the presence of CIS, and preoperative cytology were determined. If the urinary cytology was class III or greater, it was defined as positive.

BCG was scheduled for weekly administration for 6 weeks for stage Ta-T1 tumors and for 8 weeks for CIS at a dose of 80 mg of Tokyo strain or 81 mg of Connaught strain of BCG in 40 mL of saline instilled into the bladder by way of an 8 Fr urethral catheter, with retention for 1-2 hours.

These patients were assessed by urine cytology and cystoscopy every 3 months for 2 years after the initial treatment, every 6 months for the next 3 years, and then yearly thereafter. Intravenous urography or ultrasonography and/or CT scanning was used to evaluate the bladder and kidneys every 6 months for 5 years after the initial treatment, and then yearly thereafter. Histopathological extensions and grade were classified according to the TNM classification 1997[18] and WHO grading 1999[19].

The indicators of intravesical recurrence risks due to bladder biopsies were analyzed using univariate and multivariate Cox regression models. Indicators including sex, first or 2 or more TUR, grade, multiplicity, T stage, BCG therapy, presence of CIS, and enforcement of bladder biopsy were also analyzed.

## Results

The pathological characteristics of the main bladder cancers in the TUR-BT specimens of the 424 patients are listed in Table 1. One hundred and twenty-six cases (29.7%) were T1 and 144 cases (33.9%) consisted of a G3 element. Solitary tumors and primary cases were observed in 45.0% and 50.2%, respectively. In total, bladder biopsies were performed in 293 patients. Biopsy from suspicious-appearing urothelium and multiple biopsies from normal-appearing urothelium were performed in 59 patients (Table 2) and in 234 patients (Table 3), respectively. Both biopsies were performed in 34 patients.

**Table 1:** Tumor characteristics of the visible tumors resected (TUR specimens) in 424 patients

Primary pathology in TUR	Total (%)
TaG1	33 (7.8)

TaG2	189 (44.6)

TaG3	62 (14.6)

T1G1	5 (1.2)

T1G2	39 (9.2)

T1G3	82 (19.3)

CIS	14 (3.3)

Solitary	191 (45.0)

Multiple	233 (55.0)

Primary	213 (50.2)

Recurrence	211 (49.8)

Total	424 (100)

**Table 2:** Pathological results of biopsy from suspicious-appearing urothelium in 59 cases

TUR specimen (n)	Biopsy from suspicious-appearing urothelium							Total (%)
		
	TaG1	TaG2	TaG3	T1G1	T1G2	T1G3	CIS	
TaG1(1)	0	0	0	0	0	0	0	0(0)

TaG2(23)	0	5	0	0	0	0	2	7(30.4)

TaG3(13)	0	0	1	0	0	0	3	4(30.8)

T1G1(0)	0	0	0	0	0	0	0	0(0)

T1G2(3)	0	0	0	0	1	0	1	2(66.7)

T1G3(12)	0	0	2	0	0	1	3	6(50.0)

CIS(7)	0	0	0	0	0	0	4	4(57.1)

Total(59)	0	5	3	0	1	1	13	23(39.0)

Positive cytology(32)	0	4	2	0	1	1	8	16(50.0)

Negative cytology(27)	0	1	1	0	0	0	5	7(25.9)

**Table 3:** Pathological results of multiple biopsies from normal-appearing urothelium in 234 cases

TUR specimen (n)	Multiple biopsies from normal-appearing urothelium							Total (%)
		
	TaG1	TaG2	TaG3	T1G1	T1G2	T1G3	CIS	
TaG1(20)	0	0	0	0	0	0	0	0(0)

TaG2(101)	1	3	0	0	0	0	0	4(4.0)

TaG3(34)	0	0	5	0	0	0	3	8(23.5)

T1G1(4)	0	0	0	0	0	0	0	0(0)

T1G2(22)	0	0	0	0	1	0	0	1(4.5)

T1G3(48)	0	1	3	0	1	2	2	9(18.8)

CIS(5)	0	0	0	0	0	0	4	4(80.0)

Total(234)	1	4	8	0	2	2	9	26(11.1)

Positive cytology(88)	1	1	6	0	0	2	8	18(20.5)

Negative cytology (146)	0	3	2	0	2	0	1	8(5.5)

The pathological results of biopsies from suspicious-appearing urothelium in 59 patients are shown in Table 2. No tumor was found in 36 patients (61.0%), and urothelial bladder cancer was observed in 23 patients (39.0%). In the biopsies, TaG2 tumor was observed in 5 patients, TaG3 tumor in 3 patients, T1G2 tumor in 1 patient, T1G3 tumor in 1 patient, and a CIS lesion in 13 patients. No T2 tumor was detected in the biopsies. Of the 59 cases, 45.7% were urinary cytology negative. No upstaging or upgrading cases were found in these patients based on these biopsy results. Nine cases with visible tumors resected (TaG2:2, TaG3:3, T1G2:1, T1G3:3) had CIS only in the biopsies from suspicious-appearing urothelium. Urinary cytology was negative in 3 of the 9 cases who had CIS only in biopsies from suspicious-appearing urothelium (Table4). Four cases were recognized as multiple tumors based on the additional information obtained from the biopsies. Urinary cytology was negative in 2 of the 4 cases who recognized as multiple tumors based on the additional biopsies from suspicious-appearing urothelium. Ultimately, 13 cases (22.0%) with TUR plus biopsies from suspicious-appearing urothelium were diagnosed as having more malignant features, including CIS and multiplicity, than that in the TUR specimen alone. Based on the biopsies from suspicious-appearing urothelium, therapy was not altered in any of the 13 patients.

**Table 4:** CIS detected only in bladder biopsy specimen

	TUR-BT specimen	Number of Pt
biopsies from suspicious-appearing urothelium & positive cytology	pTaG2	2
	pTaG3	1
	pT1G2	1
	pT1G3	2

multiple biopsies from normal-appearing urothelium & positive cytology	pTaG3	3
	pT1G3	2

biopsies from suspicious-appearing urothelium & negative cytology	pTaG3	2
	pT1G3	1

multiple biopsies from normal-appearing urothelium & negative cytology		0

total		14

Pt: patient

In 34 patients who underwent biopsies from suspicious-appearing urothelium and multiple biopsies from normal-appearing urothelium, no tumor was observed in the biopsies from normal-appearing urothelium.

The pathological results for 234 patients who received multiple biopsies from normal-appearing urothelium alone with TUR-BT are summarized in Table 3. No tumor was found in the multiple biopsies from normal-appearing urothelium of 208 patients (88.9%). Urothelial bladder cancer was observed in 26 patients (11.1%, TaG1: 1, TaG2: 4, TaG3: 8, T1G2: 2, T1G3: 2, CIS: 9). Of these 234 cases, 62.4% were urinary cytology negative. No T2 tumor was detected in the biopsies. No upstaging or upgrading cases were found. Five cases with visible tumors (TaG3: 3, T1G3: 2) had CIS only in the multiple biopsies from normal-appearing urothelium (Table 4). In these 5 cases, no patient had a negative cytology result. Three cases were recognized as multiple tumors based on the addition of multiple biopsies from normal-appearing urothelium with TUR (T1G3: 3). In these 3 cases, no patient had a negative cytology result. Ultimately, 8 cases (3.4%) with TUR plus multiple biopsies from normal-appearing urothelium were diagnosed as having more malignant features, including CIS and multiplicity, than the TUR specimens alone. Altogether, due to the biopsies from suspicious-appearing urothelium, therapy was not altered in any of the 8 patients.

**Figure 1:**
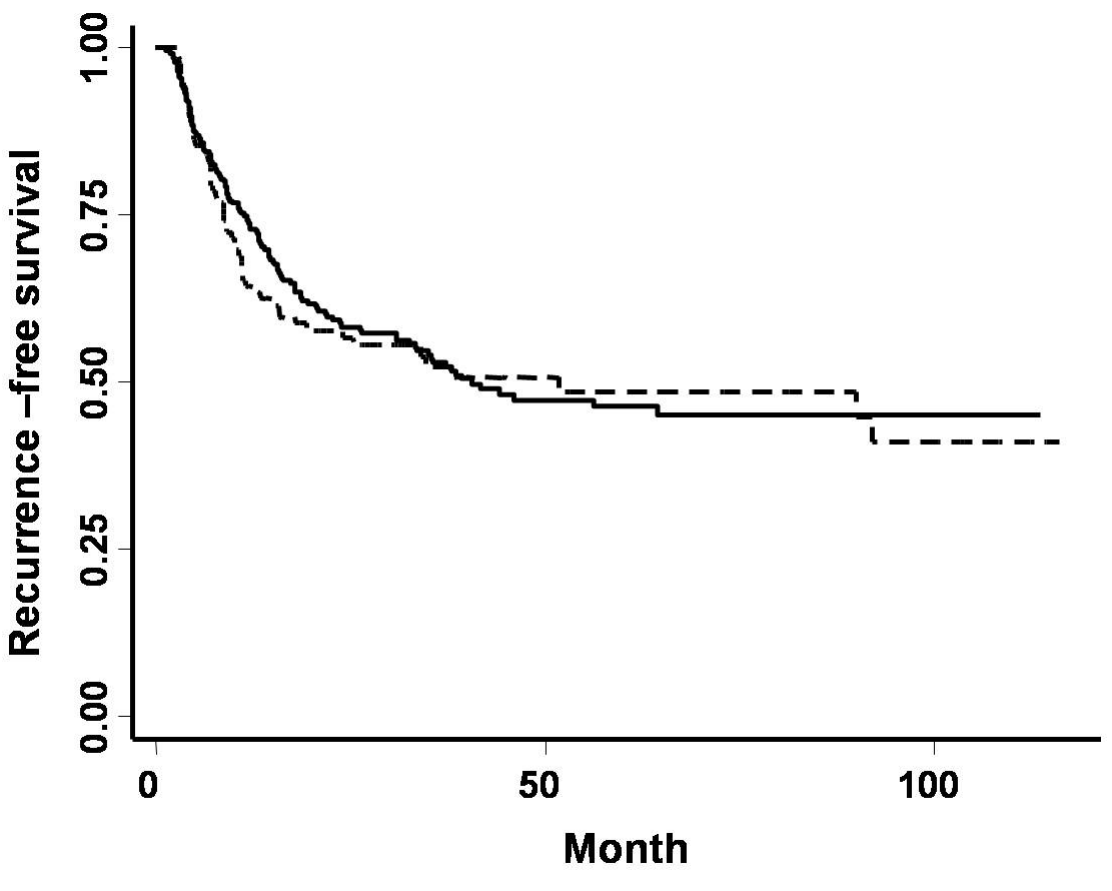
**The Kaplan-Meier curve of tumor recurrence in patients with or without bladder biopsy (p = 0.694)**. Continuous line; bladder Bx (+), Dashed line; bladder Bx (-)

We stated the differences in the backgrounds of patients who underwent biopsy and those who did not (Table 5). As shown in Table 5, age, sex, solitary or multiple, stage, grade, and with or without BCG therapy were not different between the patients who did and did not undergo biopsies. The patients with primary tumor underwent bladder biopsies more frequently than the patients with recurrence tumor. The results of analysis of factors affecting intravesical recurrence using Cox's proportional hazards model are shown in Table 6. Univariate analysis showed that instillation of BCG, tumor grade, tumor stage, multiplicity, and recurrent cases were significant prognostic factors for intravesical recurrence (p < 0.05). Multivariate analysis demonstrated that instillation of BCG, tumor stage, multiplicity, and recurrent cases were significant prognostic factors for tumor recurrence. Whether or not a bladder biopsy was performed was not a significant factor in either the univariate or multivariate analysis. As shown in the Figure 1, the recurrence rate was not different between the patients who did or did not undergo bladder biopsy either at an early time point or later time point.

**Table 5:** The comparison of clinical characteristics and primary pathology between TUR-BT with or without bladder biopsy

		Without bladder Bx (TUR-BT only)	With bladder Bx	P value
Sex				
	Male	114	235	0.089
	Female	17	58	
Age				
	< 65	43	108	0.473
	≥65	88	185	
Tumor				
	Solitary	57	134	0.670
	Multiple	74	159	
TUR-BT				
	Primary	42	171	< 0.001
	Recurrence	89	122	
Stage				
	Ta	92	192	0.542
	T1	37	89	
Grade				
	G1	13	25	0.702
	G2, G3	116	256	
Post BCG				
	+	43	120	0.112
	-	88	173	

Bx; biopsy

**Table 6:** Analysis of factors affecting intravesical recurrence

	Univariate analysis (p value)	Multivariate analysis (p value)	Standard error	Hazards ratio
male vs. female	0.6452			
primary vs. recurrence	0.0001	0.0003	0.153	1.737
solitary vs. multiple	0.0020	< 0.0001	0.155	1.853
G1/2 vs. G3	0.0405			
Ta vs. T1	0.0344	0.0009	0.156	1.678
with or without BCG Tx	< 0.0001	< 0.0001	0.159	2.143
presence or absence of CIS	0.2414			
with or without bladder Bx	0.6943			

TUR: transurethral resection

Tx: treatment

Bx: biopsy

## Discussion

The clinical significance of multiple biopsies from normal-appearing urothelium is still controversial. Several studies have demonstrated the usefulness of multiple biopsies from normal-appearing urothelium[6,10,13,20]. Recently, May et al. reported that the normal-appearing urothelium of 128 patients (12.4%) with non-muscle-invasive bladder carcinoma had cancer tissue[6]. Among these 128 patients, 58.6% were recognized as upstaging and therapy was altered in 54.6% based on the results of the multiple biopsies from normal-appearing urothelium in addition to the results of the TUR specimen. The authors concluded that multiple biopsies from normal-appearing urothelium had a strong impact on the choice of the therapeutic strategy for the patient. In contrast, some investigators believe that multiple biopsies from normal-appearing urothelium are unnecessary because of the low incidence of positive biopsy[2,4]. One of the most important studies on taking multiple biopsies from normal-appearing urothelium in non-muscle-invasive bladder cancer was performed by the European Organization for Research and Treatment of Cancer (EORTC)[4]. In the EORTC No. 30863-protocol, 393 patients with solitary and Ta or T1 tumors (low risk tumors) underwent multiple biopsies from normal-appearing urothelium and CIS was detected in only 6 patients (1.5%). In the EORTC No. 30911-protocol, multiple biopsies from normal-appearing urothelium were taken from 602 patients with multiple or recurrent Ta or T1 tumors (intermediate and high risk tumors), and 21 patients (3.5%) showed CIS in multiple biopsies from normal-appearing urothelium. An unexpected invasive tumor (T2) in a biopsy specimen was found in only one patient in this series. The EORTC results suggest that performing multiple biopsies from normal-appearing urothelium during TUR will not help with respect to staging and selecting adjuvant therapy after transurethral resection because of the low incidence of CIS and cases in which the therapy was altered. While the outcome of urinary cytology has great impact on the decision about performing the biopsies and determination of the stage and/or grade of bladder cancer, the results of urinary cytology were not mentioned in their reports.

In the present study, cancer tissue was detected in the multiple biopsies from normal-appearing urothelium in 26 patients. No up-staging cases were found. We also focused on tumor grading in this study. In these cases, no up-grading was found. Furthermore, all 5 CIS cases that were only detected in the multiple biopsies from normal-appearing urothelium had positive cytology results. Three cases were recognized as multiple tumors by also performing multiple biopsies from normal-appearing urothelium. These three cases had positive cytology results. In total, 8 cases were recognized as having more malignant features, including CIS and multiplicity, due to the biopsy results. As all of these cases had visible T1 or high grade or multiple tumors in the TUR specimen, they were treated with BCG instillation according to the TUR results. Therefore, therapy was not altered based on the biopsy results. Furthermore, no case with a T2 specimen was found in the multiple biopsies from normal-appearing urothelium. Taken together, multiple biopsies from normal-appearing urothelium did not provide any further information to assist with the treatment decision. Thus, we conclude that multiple biopsies from normal-appearing urothelium in negative cytology cases would not be of any real benefit.

In our study, biopsies from suspicious-appearing urothelium were performed in 59 cases, and cancer tissue (no up-staging case and up-grading were found) was detected in 23 patients (38.9%) and CIS was detected in 13 patients (22.0%). Nine cases had CIS only in the biopsies from suspicious-appearing urothelium. Of these 9 cases, 3 patients had negative cytology results. Altogether, 13 cases who underwent biopsies from suspicious-appearing urothelium were diagnosed as having more malignant features than that in the TUR specimen alone. However, almost all of these cases had visible T1 or high grade or multiple tumors in the TUR specimen. Since this was the same result as for multiple biopsies from normal-appearing urothelium, a change in clinical treatment based on the biopsies from suspicious-appearing urothelium was not undertaken in these 13 cases in our hospital.

The bladder mucosa after intravesically administered BCG or chemotherapy mimics the irregular carcinomatous mucosa. In our population, 6 patients underwent biopsy from suspicious-appearing urothelium within 6 months after BCG therapy. Three of these patients had positive results (2 patients; CIS, 1 patient; Ta on the biopsy specimen). Therefore, the other 3 patients had no malignancy in their biopsy specimens. Two patients underwent biopsy of suspicious-appearing urothelium within 6 months after intravesical chemotherapy. These patients had no malignancy in their biopsy specimens.

The EAU guideline states that, except for a papillary tumor, if the rest of the bladder mucosa appears normal and if urine cytology is negative, it is not advisable to routinely perform multiple biopsies from normal-appearing urothelium because the likelihood of detecting CIS is extremely low and the choice of adjuvant intravesical therapy is not influenced by the biopsy result. This recommendation of the EAU guideline is based on the EORTC study[1-4]. However, this study did not fully analyze the relationship between urine cytology and CIS detection. In our series, all CIS cases detected only in the multiple biopsies from normal-appearing urothelium had positive cytology results. On the other hand, the positive rate for detecting CIS (22.0%) in biopsies from suspicious-appearing urothelium is significantly higher than that of multiple biopsies from normal-appearing urothelium (3.8%). Thus, if the patients have suspicious-appearing urothelium or positive cytology results, we believe that bladder biopsy may be useful for detecting CIS because of staging properties. This result supported the recommendations of the EAU guideline.

Several reports have suggested that bladder biopsy provokes implantation of tumor cells in biopsy sites[6,7,10,14-17]. Levi et al. reported that cold-cup bladder biopsy was highly associated with CIS recurrence compared to hot wire loop biopsy, and thus concluded that bladder biopsy provokes implantation of neoplastic cells[15]. In contrast, our univariate and multivariate analyses demonstrated that performing bladder biopsy in addition to TUR-BT was not a significant risk factor for tumor recurrence. As shown in Figure 1, the recurrence rate was not different between patients with or without bladder biopsy at either an early time point or late time point.

There are several limitations to our study. First, the results were analyzed in a retrospective fashion. There is also a lack of reproducibility in the determination of histological aberrations such as dysplasia, metaplasia, and hyperplasia in the biopsy specimens. Therefore, the interpretation of histological abnormalities in the bladder biopsies is somewhat controversial. There is a significant bias in regard to our data taken from non-standardized reports from multiple pathologists. The high inter- and intra-pathologist variation indicates that these alterations are difficult to interpret and provide no reliable clinical value for the urologist. However, at our institution, all pathological and cytological specimens were carefully reviewed by uro-pathologists and well trained cytologists. Another limitation is that a second TUR was not performed in the diagnosis and treatment of bladder tumor in our cases because they were not routinely performed at our institution. No maintenance BCG therapy or immediate intravesical chemotherapy was performed in the study period.

## Conclusions

Bladder cancer was detected in 26 of 234 cases (11.1%) by performing multiple biopsies from normal-appearing urothelium in addition to TUR-BT. In total, 8 cases were recognized as having more malignant features, including CIS and multiplicity, due to the biopsy results. The 8 cases had all positive cytology results. The therapeutic decision was not altered in these 8 cases. Thus, our findings suggest that multiple biopsies from normal-appearing urothelium would not be of benefit and are therefore unnecessary in patients with negative cytology results. On the other hand, the positive rate for detecting CIS (N = 9, 22.0%) in biopsies from suspicious-appearing urothelium was relatively high. Meanwhile, 3 patients with CIS only detected in the biopsies from suspicious-appearing urothelium had negative cytology results. In patients with negative cytology results, suspicious-appearing urothelium would be needed in order to detect CIS because of staging properties. This result supported the recent EAU guideline.

## Competing interests

The authors declare that they have no competing interests.

## Authors' contributions

MH, KM and MM formulated database. MM performed the initial analyses and drafted the first manuscript. All authors assisted in the analysis and interpretation of data. EK conceived of the study, and participated in its design and coordination and helped to draft the manuscript. All authors read and approved the final manuscript.

## Pre-publication history

The pre-publication history for this paper can be accessed here:

http://www.biomedcentral.com/1471-2490/10/12/prepub
